# Supplementary material for: Association between metabolic syndrome and 13 types of cancer in Catalonia: A matched case-control study
Source: PLoS One. 2022 Mar 4;17(3):e0264634. doi: 10.1371/journal.pone.0264634 (PMC8896701; doi:10.1371/journal.pone.0264634)
Supplement: S1 Table — (DOCX) [file pone.0264634.s001.docx]

**S1Table. MS components combination with cancer cases and matched controls**

|  | All Cases n(%) | All Controls n(%) |
| --- | --- | --- |
| N total | 183284 | 733136 |
| **Metabolic Syndrome** |  |  |
| No component | 46324 (25.3) | 207337 (28.3) |
| 1 component | 47573 (26.0) | 189204 (25.8) |
| Obesity (↑BMI) | 1648 (3.5) | 6662 (3.5) |
| High blood pressure (↑BP) | 38200 (80.3) | 152139 (80.4) |
| Reduced HDL cholesterol (↓HDL) | 1705 (3.6) | 6720 (3.6) |
| Elevated Triglycerides (↑TG) | 1165 (2.4) | 4497 (2.4) |
| High Glycemia (↑Gly) | 4855 (10.2) | 19186 (10.1) |
| 2 components | 36184 (19.7) | 139784 (19.1) |
| ↑BP-↑Gly | 17032 (47.1) | 64771 (46.3) |
| ↑BP-↑BMI | 8986 (24.8) | 34458 (24.7) |
| ↑BP-↓HDL | 3215 (8.9) | 13266 (9.5) |
| ↑BP-↑TG | 2979 (8.2) | 11718 (8.4) |
| ↑Gly-↑TG | 1107 (3.1) | 4293 (3.1) |
| ↑Gly-↓HDL | 1111 (3.1) | 4063 (2.9) |
| ↑Gly-↑BMI | 744 (2.1) | 3109 (2.2) |
| ↓HDL-↑TG | 613 (1.7) | 2511 (1.8) |
| ↑BMI-↓HDL | 250 (0.7) | 970 (0.7) |
| ↑BMI-↑TG | 147 (0.4) | 625 (0.4) |
| MS | 53203 (29.0) | 196811 (26.8) |
| ↑BP-↑BMI-↑Gly | 9324 (17.5) | 34831 (17.7) |
| ↑BP-↑BMI-↓HDL-↑Gly-↑TG | 7995 (15.0) | 28548 (14.5) |
| ↑BP-↓HDL-↑Gly-↑TG | 6219 (11.7) | 22879 (11.6) |
| ↑BP-↑Gly-↑TG | 6147 (11.6) | 22348 (11.4) |
| ↑BP-↓HDL-↑Gly | 5439 (10.2) | 20713 (10.5) |
| ↑BP-↑BMI-↑Gly-↑TG | 5281 (9.9) | 19370 (9.8) |
| ↑BP-↑BMI-↓HDL-↑Gly | 5106 (9.6) | 18110 (9.2) |
| ↑BP-↓HDL-↑TG | 1905 (3.6) | 7602 (3.9) |
| ↑BP-↑BMI-↓HDL | 1481 (2.8) | 5744 (2.9) |
| ↑BP-↑BMI-↑TG | 1268 (2.4) | 5031 (2.6) |
| ↑BP-↑BMI-↓HDL-↑TG | 1199 (2.3) | 4538 (2.3) |
| ↓HDL-↑Gly-↑TG | 790 (1.5) | 2962 (1.5) |
| ↑BMI-↓HDL-↑Gly | 314 (0.6) | 1272 (0.6) |
| ↑BMI-↓HDL-↑Gly-↑TG | 335 (0.6) | 1233 (0.6) |
| ↑BMI-↑Gly-↑TG | 272 (0.5) | 1106 (0.6) |
| ↑BMI-↓HDL-↑TG | 128 (0.2) | 524 (0.3) |
