# Supplementary material for: Association between metabolic syndrome and 13 types of cancer in Catalonia: A matched case-control study
Source: PLoS One. 2022 Mar 4;17(3):e0264634. doi: 10.1371/journal.pone.0264634 (PMC8896701; doi:10.1371/journal.pone.0264634)
Supplement: S2 Table — (DOCX) [file pone.0264634.s002.docx]

**S2 Table. Characteristics of the controls according to Metabolic Syndrome**

|  | Metabolic Syndrome | | | | |
| --- | --- | --- | --- | --- | --- |
|  | 0 | 1 | 2 | MS (≥3) | P TREND^1^ |
| N total | 207337 | 189204 | 139784 | 196811 |  |
| **Age** mean (SD) | 60.6 (12.3) | 68.6 (12.3) | 70.5 (11.4) | 71.4 (10.3) | **<0.001^2^** |
| Median (IQR) | 59 (51-69) | 69 (60-78) | 71 (63-79) | 72 (64-79) | **<0.001^3^** |
| **Sex** |  |  |  |  |  |
| Men | 105144 (50.7) | 106341 (56.2) | 84519 (60.5) | 116928 (59.4) |  |
| Women | 102193 (49.3) | 82863 (43.8) | 55265 (39.5) | 79883 (40.6) | **<0.001** |
| **Nationality** |  |  |  |  |  |
| Spanish | 184059 (88.8) | 181449 (95.9) | 135225 (96.7) | 191155 (97.1) |  |
| Non-Spanish | 23278 (11.2) | 7755 (4.1) | 4559 (3.3) | 5656 (2.9) | **<0.001** |
| **MEDEA index** |  |  |  |  |  |
| Quintile 1 | 41234 (22.8) | 34711 (20.7) | 19182 (15.3) | 22299 (12.5) |  |
| Quintile 2 | 29973 (16.6) | 27754 (16.5) | 20125 (16) | 26802 (15) |  |
| Quintile 3 | 27137 (15) | 25778 (15.4) | 20379 (16.2) | 29462 (16.5) |  |
| Quintile 4 | 25743 (14.2) | 23867 (14.2) | 20194 (16.1) | 30797 (17.2) |  |
| Quintile 5 | 22512 (12.5) | 19919 (11.9) | 17376 (13.8) | 28759 (16.1) |  |
| Rural | 34122 (18.9) | 35798 (21.3) | 28343 (22.6) | 40668 (22.7) | **<0.001** |
| **Smoking status** |  |  |  |  |  |
| Never smoker | 29628 (60.6) | 67852 (67.1) | 61920 (66.1) | 89519 (62.5) |  |
| Ex-smoker | 6117 (12.5) | 15592 (15.4) | 18412 (19.7) | 35107 (24.5) |  |
| Smoker | 13164 (26.9) | 17670 (17.5) | 13340 (14.2) | 18508 (12.9) |  |
| **Alcohol intake** |  |  |  |  | **<0.001** |
| No consumption | 21781 (64.1) | 54422 (62.9) | 56306 (60.8) | 100829 (63.5) |  |
| Low consumption | 11123 (32.7) | 29417 (34) | 33442 (36.1) | 53705 (33.8) |  |
| High consumption | 1064 (3.1) | 2652 (3.1) | 2889 (3.1) | 4362 (2.7) | **<0.001** |
| **Hormonal therapy (women postmenopausia)** |  |  |  |  |  |
| No consumption | 49638 (95.0) | 59565 (92.7) | 43611 (92.9) | 68044 (93.6) |  |
| Consumption | 2589 (5.0) | 4708 (7.3) | 3326 (7.1) | 4627 (6.4) | **<0.001** |
| **Paracetamol** |  |  |  |  |  |
| No consumption | 190784 (92.0) | 139383 (73.7) | 90129 (64.5) | 109715 (55.7) |  |
| Consumption | 16553 (8.0) | 49821 (26.3) | 49655 (35.5) | 87096 (44.3) | **<0.001** |
| **Acetylsalicylic acid (ASA)** |  |  |  |  |  |
| No consumption | 202393 (97.6) | 160601 (84.9) | 109913 (78.6) | 137776 (70.0) |  |
| Consumption | 4944 (2.4) | 28603 (15.1) | 29871 (21.4) | 59035 (30.0) | **<0.001** |
| **Ibuprofen** |  |  |  |  |  |
| No consumption | 193802 (93.5) | 163239 (86.3) | 116220 (83.1) | 160319 (81.5) |  |
| Consumption | 13535 (6.5) | 25965 (13.7) | 23564 (16.9) | 36492 (18.5) | **<0.001** |
| **Chronic Hepatitis** |  |  |  |  |  |
| No hepatitis | 205910 (99.3) | 186558 (98.6) | 137657 (98.5) | 193911 (98.5) |  |
| Hepatitis B | 316 (0.2) | 520 (0.3) | 472 (0.3) | 806 (0.4) |  |
| Hepatitis C | 1088 (0.5) | 2087 (1.1) | 1625 (1.2) | 2053 (1) |  |
| Other/unspecified hepatitis | 23 (0) | 39 (0) | 30 (0) | 41 (0) | **<0.001** |
| **Menarche age** mean (SD) | 12.8 (1.5) | 12.8 (1.6) | 12.7 (1.6) | 12.6 (1.6) | **<0.001** |
| median (IQR) | 13 (12-14) | 13 (12-14) | 13 (12-14) | 13 (12-14) | **<0.001** |
| **Menopause** |  |  |  |  |  |
| No | 49966 (48.9) | 18590 (22.4) | 8328 (15.1) | 7212 (9.0) |  |
| Yes | 52227 (51.1) | 64273 (77.6) | 46937 (84.9) | 72671 (91.0) | **<0.001** |

^1^Unless otherwise specified, P value was derived from the Chi-Square Test.

^2^ANOVA test. ^3^Mann-Whitney U test

SD, Standard Deviation; IQR, Inter Quartile Range, MS, Metabolic Syndrome
