# Supplementary material for: Association between metabolic syndrome and 13 types of cancer in Catalonia: A matched case-control study
Source: PLoS One. 2022 Mar 4;17(3):e0264634. doi: 10.1371/journal.pone.0264634 (PMC8896701; doi:10.1371/journal.pone.0264634)
Supplement: S3 Table — Adjusted ORs and 95% CI. Multiple imputation by chained equations with 20 imputed datasets were applied to outcomes and covariates. Models adjusted by age, medea, tobacco, alcohol, nationality. aConsider two measures of parameters separated at least by 2 weeks (maximum 1 year) to ensure that the patient has that pathological component of MS. bUsing Waist circumference instead of one measure of BMI. There were 157,872 (86.1%) missing values in the case group and 636,984 (86.9%) missing values in the control group. cAlso adjusted by hepatitis and others liver disease. dThe model does not converge when using Waist circumference instead of one measure od BMI, because the number of observations in this model are very low. 1Wald test. 2P-Trend. OR, odds ratio; CI, confidence interval. (DOCX) [file pone.0264634.s003.docx]

**S3 Table. Analysis of those patients exposed to a single value of the pathological component versus those who present 2 measures and analysis considering waist circumference instead of BMI. Adjusted ORs and 95% CI**

|  | **1 Measure** | | |  | **2 Measures^a^** | | |  | **Waist circumference^b^** | | |
| --- | --- | --- | --- | --- | --- | --- | --- | --- | --- | --- | --- |
|  | OR | 95%CI | P-value |  | OR | 95%CI | P-value |  | OR | 95%CI | P-value |
| **DIGESTIVE** |  |  |  |  |  |  |  |  |  |  |  |
| **Colorectal** |  |  |  |  |  |  |  |  |  |  |  |
| No component | 1.00 |  | <0.001^1^ |  | 1.00 |  | <0.001^1^ |  | 1.00 |  | <0.001^1^ |
| 1 component | 1.09 | (1.05-1.13) | <0.001^2^ |  | 1.11 | (1.08-1.15) | <0.001^2^ |  | 1.35 | (1.00-1.83) | <0.001^2^ |
| 2 components | 1.17 | (1.12-1.21) |  |  | 1.22 | (1.18-1.27) |  |  | 1.34 | (1.00-1.81) |  |
| MS | 1.28 | (1.23-1.32) |  |  | 1.32 | (1.27-1.37) |  |  | 1.50 | (1.12-2.01) |  |
| **Liver^c^** |  |  |  |  |  |  |  |  |  |  |  |
| No component | 1.00 |  | <0.001^1^ |  | 1.00 |  | <0.001^1^ |  | 1.00 |  | <0.001^1^ |
| 1 component | 1.33 | (1.20-1.47) | <0.001^2^ |  | 1.51 | (1.38-1.65) | <0.001^2^ |  | 0.95 | (0.44-2.07) | <0.001^2^ |
| 2 components | 1.59 | (1.43-1.77) |  |  | 2.10 | (1.89-2.32) |  |  | 1.29 | (0.62-2.71) |  |
| MS | 1.93 | (1.74-2.14) |  |  | 2.35 | (2.10-2.62) |  |  | 1.76 | (0.85-3.64) |  |
| **Pancreas** |  |  |  |  |  |  |  |  |  |  |  |
| No component | 1.00 |  | <0.001^1^ |  | 1.00 |  | <0.001^1^ |  | 1.00 |  | <0.001^1^ |
| 1 component | 1.26 | (1.15-1.39) | <0.001^2^ |  | 1.21 | (1.11-1.31) | <0.001^2^ |  | 0.96 | (0.40-2.29) | <0.001^2^ |
| 2 components | 1.52 | (1.37-1.68) |  |  | 1.56 | (1.42-1.72) |  |  | 1.13 | (0.49-2.63) |  |
| MS | 1.79 | (1.63-1.98) |  |  | 1.78 | (1.61-1.97) |  |  | 1.55 | (0.67-3.56) |  |
| **GYNECOLOGICAL** |  |  |  |  |  |  |  |  |  |  |  |
| **Breast**  **Pre-Menopause** |  |  |  |  |  |  |  |  |  |  |  |
| No component | 1.00 |  | <0.001^1^ |  | 1.00 |  | 0.243^1^ |  | 1.00 |  | 0.343^1^ |
| 1 component | 1.03 | (0.98-1.08) | <0.001^2^ |  | 0.98 | (0.93-1.03) | 0.064^2^ |  | 0.90 | (0.63-1.28) | 0.075^2^ |
| 2 components | 0.94 | (0.88-1.01) |  |  | 0.91 | (0.82-1.01) |  |  | 0.87 | (0.62-1.23) |  |
| MS | 0.85 | (0.78-0.92) |  |  | 0.94 | (0.82-1.08) |  |  | 0.76 | (0.55-1.06) |  |
| **Breast**  **Post-Menopause** |  |  |  |  |  |  |  |  |  |  |  |
| No component | 1.00 |  | <0.001^1^ |  | 1.00 |  | <0.001^1^ |  | 1.00 |  | 0.308^1^ |
| 1 component | 1.04 | (1-1.09) | <0.001^2^ |  | 1.08 | (1.04-1.12) | <0.001^2^ |  | 1.38 | (0.93-2.04) | 0.193^2^ |
| 2 components | 1.06 | (1.02-1.11) |  |  | 1.11 | (1.06-1.16) |  |  | 1.37 | (0.94-2.00) |  |
| MS | 1.10 | (1.06-1.15) |  |  | 1.13 | (1.08-1.19) |  |  | 1.41 | (0.97-2.05) |  |
| **Endometrial**  **Pre-Menopause** |  |  |  |  |  |  |  |  |  |  |  |
| No component | 1.00 |  | <0.001^1^ |  | 1.00 |  | <0.001^1^ |  | 1.00 |  | 0.029^1^ |
| 1 component | 1.26 | (1.06-1.50) | <0.001^2^ |  | 1.45 | (1.22-1.71) | <0.001^2^ |  | 3.34 | (0.65-17.2) | 0.003^2^ |
| 2 components | 1.68 | (1.36-2.08) |  |  | 1.99 | (1.51-2.63) |  |  | 4.30 | (0.85-21.7) |  |
| MS | 2.14 | (1.74-2.65) |  |  | 2.61 | (1.94-3.53) |  |  | 7.14 | (1.47-34.7) |  |
| **Endometrial**  **Post-Menopause** |  |  |  |  |  |  |  |  |  |  |  |
| No component | 1.00 |  | <0.001^1^ |  | 1.00 |  | <0.001^1^ |  | 1.00 |  | <0.001^1^ |
| 1 component | 1.34 | (1.20-1.50) | <0.001^2^ |  | 1.34 | (1.22-1.48) | <0.001^2^ |  | 1.66 | (0.55-5.01) | <0.001^2^ |
| 2 components | 1.67 | (1.49-1.88) |  |  | 1.69 | (1.51-1.88) |  |  | 1.88 | (0.65-5.46) |  |
| MS | 2.46 | (2.20-2.74) |  |  | 2.42 | (2.17-2.71) |  |  | 2.91 | (1.02-8.32) |  |

(cont.).

|  | **1 Measure** | | |  | **2 Measures^a^** | | |  | **Waist circumference^b^** | | |
| --- | --- | --- | --- | --- | --- | --- | --- | --- | --- | --- | --- |
|  | OR | 95%CI | P-value |  | OR | 95%CI | P-value |  | OR | 95%CI | P-value |
| **UROLOGICAL** |  |  |  |  |  |  |  |  |  |  |  |
| **Bladder** |  |  |  |  |  |  |  |  |  |  |  |
| No component | 1.00 |  | <0.001^1^ |  | 1.00 |  | <0.001^1^ |  | 1.00 |  | 0.003^1^ |
| 1 component | 1.21 | (1.15-1.27) | <0.001^2^ |  | 1.18 | (1.05-1.32) | <0.001^2^ |  | 1.41 | (0.91-2.16) | <0.001^2^ |
| 2 components | 1.29 | (1.22-1.36) |  |  | 1.54 | (1.35-1.76) |  |  | 1.54 | (1.01-2.34) |  |
| MS | 1.41 | (1.34-1.48) |  |  | 1.87 | (1.62-2.15) |  |  | 1.68 | (1.11-2.55) |  |
| **Kidney** |  |  |  |  |  |  |  |  |  |  |  |
| No component | 1.00 |  | <0.001^1^ |  | 1.00 |  | <0.001^1^ |  | 1.00 |  | 0.006^1^ |
| 1 component | 1.42 | (1.31-1.54) | <0.001^2^ |  | 1.64 | (1.45-1.85) | <0.001^1^ |  | 2.21 | (1.06-4.61) | 0.001^2^ |
| 2 components | 1.46 | (1.33-1.59) |  |  | 1.99 | (1.72-2.31) |  |  | 2.42 | (1.18-4.95) |  |
| MS | 1.84 | (1.69-2.00) |  |  | 2.23 | (1.92-2.60) |  |  | 2.80 | (1.38-5.68) |  |
| **Prostate** |  |  |  |  |  |  |  |  |  |  |  |
| No component | 1.00 |  | <0.001^1^ |  | 1.00 |  | <0.001^1^ |  | 1.00 |  | 0.004^1^ |
| 1 component | 1.15 | (1.11-1.19) | 0.991^2^ |  | 1.14 | (1.11-1.18) | 0.659^2^ |  | 0.80 | (0.61-1.04) | 0.001^2^ |
| 2 components | 1.14 | (1.10-1.19) |  |  | 1.10 | (1.06-1.14) |  |  | 0.75 | (0.58-0.96) |  |
| MS | 1.02 | (0.98-1.06) |  |  | 0.96 | (0.91-1.00) |  |  | 0.70 | (0.55-0.90) |  |
| **HEMATOLOGICAL** |  |  |  |  |  |  |  |  |  |  |  |
| **Hodgkin^d^** |  |  |  |  |  |  |  |  |  |  |  |
| No component | 1.00 |  | 0.462^1^ |  | 1.00 |  | 0.070^1^ |  | --- | --- | --- |
| 1 component | 0.92 | (0.63-1.34) | 0.049^2^ |  | 1.00 | (0.79-1.26) | 0.020^2^ |  | --- | --- | --- |
| 2 components | 1.28 | (0.82-1.99) |  |  | 1.33 | (0.97-1.81) |  |  | --- | --- |  |
| MS | 1.19 | (0.78-1.82) |  |  | 1.45 | (1.02-2.05) |  |  | --- | --- |  |
| **Non-Hodgkin** |  |  |  |  |  |  |  |  |  |  |  |
| No component | 1.00 |  | <0.001^1^ |  | 1.00 |  | 0.006^1^ |  | 1.00 |  | 0.565^1^ |
| 1 component | 1.12 | (1.01-1.24) | <0.001^2^ |  | 1.07 | (0.97-1.18) | <0.001^2^ |  | 1.10 | (0.47-2.59) | 0.920^2^ |
| 2 components | 1.24 | (1.10-1.39) |  |  | 1.14 | (1.01-1.29) |  |  | 1.36 | (0.60-3.06) |  |
| MS | 1.23 | (1.10-1.38) |  |  | 1.27 | (1.10-1.45) |  |  | 1.17 | (0.53-2.59) |  |
| **Leukaemia** |  |  |  |  |  |  |  |  |  |  |  |
| No component | 1.00 |  | <0.001^1^ |  | 1.00 |  | <0.001^1^ |  | 1.00 |  | 0.562^1^ |
| 1 component | 1.23 | (1.14-1.33) | <0.001^2^ |  | 1.20 | (1.11-1.28) | <0.001^2^ |  | 1.35 | (0.61-3.00) | 0.214^2^ |
| 2 components | 1.34 | (1.22-1.46) |  |  | 1.26 | (1.15-1.37) |  |  | 1.49 | (0.69-3.24) |  |
| MS | 1.42 | (1.31-1.54) |  |  | 1.36 | (1.24-1.49) |  |  | 1.54 | (0.71-3.31) |  |
| **OTHERS** |  |  |  |  |  |  |  |  |  |  |  |
| **Lung** |  |  |  |  |  |  |  |  |  |  |  |
| No component | 1.00 |  | <0.001^1^ |  | 1.00 |  | 0.029^1^ |  | 1.00 |  | 0.044^1^ |
| 1 component | 1.09 | (1.05-1.15) | <0.001^2^ |  | 1.04 | (1.00-1.08) | 0.005^2^ |  | 0.87 | (0.59-1.28) | 0.017^2^ |
| 2 components | 1.08 | (1.02-1.13) |  |  | 1.04 | (0.99-1.10) |  |  | 0.89 | (0.61-1.29) |  |
| MS | 1.11 | (1.05-1.16) |  |  | 1.09 | (1.03-1.15) |  |  | 1.01 | (0.70-1.45) |  |
| **Thyroid** |  |  |  |  |  |  |  |  |  |  |  |
| No component | 1.00 |  | <0.001^1^ |  | 1.00 |  | <0.001^1^ |  | 1.00 |  | 0.166^1^ |
| 1 component | 1.29 | (1.15-1.45) | <0.001^2^ |  | 1.44 | (1.29-1.61) | <0.001^2^ |  | 1.14 | (0.45-2.86) | 0.055^2^ |
| 2 components | 1.57 | (1.38-1.80) |  |  | 1.49 | (1.28-1.73) |  |  | 1.07 | (0.43-2.64) |  |
| MS | 1.71 | (1.50-1.95) |  |  | 1.65 | (1.39-1.97) |  |  | 1.54 | (0.64-3.71) |  |

Multiple imputation by chained equations with 20 imputed datasets were applied to outcomes and covariates. Models adjusted by age, medea, tobacco, alcohol, nationality.

^a^Consider two measures of parameters separated at least by 2 weeks (maximum 1 year) to ensure that the patient has that pathological component of MS.

^b^Using Waist circumference instead of one measure of BMI. There were 157,872 (86.1%) missing values in the case group and 636,984 (86.9%) missing values in the control group.

^c^Also adjusted by hepatitis and others liver disease.

^d^The model does not converge when using Waist circumference instead of one measure od BMI, because the number of observations in this model are very low.

^1^Wald test. ^2^P-Trend

OR, odds ratio; CI, confidence interval
