# Supplementary material for: Association between metabolic syndrome and 13 types of cancer in Catalonia: A matched case-control study
Source: PLoS One. 2022 Mar 4;17(3):e0264634. doi: 10.1371/journal.pone.0264634 (PMC8896701; doi:10.1371/journal.pone.0264634)
Supplement: Attachment — Submitted filename: PONE-D-21-30858_reviewer.docx [file pone.0264634.s004.docx]

This article reported results MS is statistically associated with a higher risk of developing at 11 cancer types after the evaluation of 13. Moreover, they demonstrated in this manuscript that the risk of most cancers increased with the number of MS components present in an individual. Hence, the authors finally suggest that prevention strategies targeting individual components of MS could reduce the risk of several cancer types. Although the manuscript is potentially interesting, it is mostly descriptive and most of the conclusions have been published previously (see comments below).

Thus, there are major aspects that could be revised to improve the results:

- The authors should take care in the use of “recent” when they are referring to other published works since I cannot consider a recent work that has been published more than 5 years ago. An example is given, “MS was initially considered a risk factor just for cardiovascular disease. However, recent studies associate MS with a higher risk of liver, colorectal, and bladder cancer in men; and endometrial, pancreatic, colorectal, ovarian, and postmenopausal breast cancer in women”. However, the original manuscript was published in 2012(doi: 10.2337/dc12-0336).

- The authors did not give too many reasons for the selection of these 13 cancer types. The authors only said, “However, no evidence has been yet provided for the impact of MS components in other less common cancers”. Conversely, for example, lung cancer has related to some metabolic factors of metabolic syndrome. Please, I am really encouraged to provide more reasons for the selection of these cancer types.

- The author must justify the fact of comparing every case with four controls since a major number of control cases could imply a major statistical power being really important for the final results and conclusions of this study.

-In general, the manuscript needs to get a significant improvement in the discussion section. For instance, the sentence is too descriptive: “In gynecological cancers, the menopausal status was a determinant factor, especially in breast cancer. In agreement with previous studies, we observed that MS increased breast cancer risk in postmenopausal women and decreased it in premenopausal women”.

- Concerning the important limitation of not following up with the patients, the authors should clearly explain this limitation and how it could affect the results of the paper. Furthermore, the authors should provide information about when the MS component was identified such as previous to tumor diagnose (1 year or 2 years), during tumor treatment…

Minor comments:

- A sentence in the abstract is not very clear. “Adjusted conditional logistic regression models were used to estimate OR and 95% CI for the association between individual components of MS and cancer, and MS and cancer.” It seems to be confusing.

- The use of “approximately” when you are talking about the patients available in the study is not accurate for scientific work.

- The authors described in methodology “All individuals ≥ 40-99 years of age with information in the SIDIAP database between 01/01/2006 and 31/12/2017 were included. Final participants included patients with any of the 13 types of incident cancers of interest, together with their paired controls.” Please, the authors should provide the number of patients after the application of each inclusion criteria in the methodology section.

- Typing errors like “MS component..” should be corrected in the new manuscript.
